# CAV3 alleviates diabetic cardiomyopathy via inhibiting NDUFA10-mediated mitochondrial dysfunction

**DOI:** 10.1186/s12967-024-05223-6

**Published:** 2024-04-26

**Authors:** Ping Guo, Shuiqing Hu, Xiaohui Liu, Miaomiao He, Jie Li, Tingqiong Ma, Man Huang, Qin Fang, Yan Wang

**Affiliations:** 1 Division of Cardiology and Department of Internal Medicine, Tongji Hospital, Tongji Medical College, Huazhong University of Science and Technology, Wuhan, 430030 China; 2 Hubei Key Laboratory of Genetics and Molecular Mechanisms of Cardiological Disorders, Wuhan, 430030 China

**Keywords:** CAV3, Diabetic cardiomyopathy, Apoptosis, NDUFA10, Mitochondrial dysfunction

## Abstract

**Background:**

The progression of diabetic cardiomyopathy (DCM) is noticeably influenced by mitochondrial dysfunction. Variants of caveolin 3 (CAV3) play important roles in cardiovascular diseases. However, the potential roles of CAV3 in mitochondrial function in DCM and the related mechanisms have not yet been elucidated.

**Methods:**

Cardiomyocytes were cultured under high-glucose and high-fat (HGHF) conditions in vitro, and db/db mice were employed as a diabetes model in vivo. To investigate the role of CAV3 in DCM and to elucidate the molecular mechanisms underlying its involvement in mitochondrial function, we conducted Liquid chromatography tandem mass spectrometry (LC–MS/MS) analysis and functional experiments.

**Results:**

Our findings demonstrated significant downregulation of CAV3 in the cardiac tissue of db/db mice, which was found to be associated with cardiomyocyte apoptosis in DCM. Importantly, cardiac-specific overexpression of CAV3 effectively inhibited the progression of DCM, as it protected against cardiac dysfunction and cardiac remodeling associated by alleviating cardiomyocyte mitochondrial dysfunction. Furthermore, mass spectrometry analysis and immunoprecipitation assays indicated that CAV3 interacted with NDUFA10, a subunit of mitochondrial complex I. CAV3 overexpression reduced the degradation of lysosomal pathway in NDUFA10, restored the activity of mitochondrial complex I and improved mitochondrial function. Finally, our study demonstrated that CAV3 overexpression restored mitochondrial function and subsequently alleviated DCM partially through NDUFA10.

**Conclusions:**

The current study provides evidence that CAV3 expression is significantly downregulated in DCM. Upregulation of CAV3 interacts with NDUFA10, inhibits the degradation of lysosomal pathway in NDUFA10, a subunit of mitochondrial complex I, restores the activity of mitochondrial complex I, ameliorates mitochondrial dysfunction, and thereby protects against DCM. These findings indicate that targeting CAV3 may be a promising approach for the treatment of DCM.

**Supplementary Information:**

The online version contains supplementary material available at 10.1186/s12967-024-05223-6.

## Introduction

Diabetes mellitus is on the rise globally, with projections indicating that it will impact 10.9% of the world’s population by 2045 [1]. Diabetes mellitus significantly increases the risk of cardiovascular disease [2, 3]. Diabetic cardiomyopathy (DCM), a heart disease that specifically affects individuals with diabetes, is independent of other common cardiac risk factors, such as hypertension, atherosclerosis, or significant valvular disease [4–6]. It is a major complication of diabetes mellitus and a leading cause of mortality and morbidity in patients with diabetes [5, 7–9]. However, the exact pathological and molecular mechanisms of DCM remain unclear, impeding the development of effective treatments [10].

The progression of DCM is driven by multiple underlying mechanisms, such as mitochondrial dysfunction, impaired insulin signalling, oxidative stress, endoplasmic reticulum stress, and impaired regulation of mitochondrial calcium handling [11–14]. Dysfunctional mitochondria primarily contribute to the generation of reactive oxygen species (ROS) and mediate cardiomyocyte apoptosis in diabetic hearts [15–18]. While accumulating convincing evidence highlights the pivotal role of mitochondrial dysfunction in the progression of DCM [19, 20], the development of precise and effective therapeutic strategies to mitigate mitochondrial dysfunction remains an ongoing challenge.

CAV3, a member of the caveolin protein family [21, 22], is selectively and highly expressed in cardiomyocytes [23]. Previous research has provided evidence highlighting the participation of CAV3 in the development of diverse cardiovascular diseases, including cardiac hypertrophy, contractile dysfunction, myocardial ischaemia and heart failure [24–26]. Our previous studies have demonstrated that CAV3 plays an important role in protecting against arrhythmias [27]. However, there is limited research investigating the correlation between CAV3 and mitochondrial dysfunction in cardiovascular disease [28]. Specifically, the detailed mechanisms by which CAV3 is related to mitochondrial dysfunction in DCM remain to be fully understood.

Our study revealed that CAV3 plays a significant role in the development of DCM. Mechanistically, we demonstrated that CAV3 overexpression can inhibit the degradation of NDUFA10 via the lysosomal pathway. This change subsequently regulates the activity of complex I, preserves mitochondrial function, and thus protects against DCM. These findings indicate that increasing CAV3 expression to alleviate mitochondrial dysfunction could be a therapeutic approach for DCM.

## Materials and methods

### Design of the animal experiments

The animal experiments received approval from the Experimental Animal Research Committee of Tongji Medical College, Huazhong University of Science and Technology (Wuhan, China) and were performed in compliance with the Guide for the Care and Use of Laboratory Animals provided by the National Institute of Health (Bethesda, MD).

In vivo studies were performed on 8-week-old male mice on the C57BLKS (BKS) background, specifically leptin receptor-deficient (db/db) mice and wild-type (wt) mice, sourced from GemPharmatech (Nanjing, China). Mice were individually housed in transparent plastic cages, with two mice per cage, in a room that was carefully regulated to maintain a controlled temperature and a light–dark cycle of 12 h. Mice were given unlimited access to rodent chow and tap water. All mice were euthanized at 20 weeks, and their hearts were promptly dissected and rapidly frozen in liquid nitrogen for subsequent analysis.

*Experimental design 1.* To investigate the relationship between CAV3 and DCM, we administered an adeno-associated virus 9 vector expressing CAV3 (AAV9-CAV3) under the control of the cTnT promoter or control virus. The mice were randomly divided into four groups with equal numbers (n = 7 per group): (1) the wt + AAV9-Ctrl, (2) wt + AAV9-CAV3, (3) db/db + AAV9-Ctrl, and (4) db/db + AAV9-CAV3 groups. All mice were administered recombinant AAV9 via the tail vein at the age of 9 weeks and were subsequently euthanized at 20 weeks of age. Throughout the entire experiment, the body weight and food intake of the mice were documented on a weekly basis.

*Experimental design 2.* To investigate whether CAV3 alleviates diabetic cardiomyopathy by regulating NDUFA10, we administered AAV-CAV3, AAV-shNDUFA10 or control virus by tail vein injection at a planned time. Male db/db mice were randomly divided into four groups with equal numbers (n = 7 per group): (1) the db/db + AAV-Ctrl + AAV-shNC group, (2) db/db + AAV-Ctrl + AAV-shNDUFA10 group, (3) db/db + AAV-CAV3 + AAV-shNC group, and (4) db/db + AAV-CAV3 + AAV-shNDUFA10 group. At 9 weeks of age, all mice received tail vein injection of AVV9 to upregulate CAV3, and at 10 weeks of age, they were given tail vein injections of AAV9 to downregulate NDUFA10. They were euthanized at 20 weeks.

CAV3 was overexpressed in vivo with an AVV9 vector expressing CAV3, and NDUFA10 was downregulated with an AVV9 vector expressing shNDUFA10). All the vectors mentioned above were constructed by Hanbio Biotechnology Co. Ltd. (Shanghai, China). The effects of gene overexpression or interference were verified through Western blotting analysis.

### Cell culture and treatment

Human cardiomyocytes (AC16) and mouse cardiomyocytes (HL-1) were purchased from iCell Bioscience (Shanghai, China). The cells were cultured in Dullbecco’s modified Eagle’s medium (DMEM) (Keygen Biotech, Nanjing, China) supplemented with 10% foetal bovine serum. Two different media were used: normal-glucose (NG) medium (5.5 mmol/l glucose) and high-glucose-high-fatty-acid (HGHF) medium (33 mmol/l glucose and 250 μmol/l palmitate). The cells were collected after incubation at 37 °C in 5% CO_2_ for 24 h.

### Blood glucose level measurement

A small incision was made at the tip of the tail vein after sterilizing it with an alcohol-soaked sponge. The first drop of blood was discarded, and the second drop of blood was used to measure blood glucose levels using a glucose metre (Roche Diagnostics, Mannheim, Germany).

### Fasting blood glucose (FBG) level measurement

The blood glucose levels of mice in each group were measured on the 1st day of the experiment and every 2 weeks afterward. Mice were fasted for 8 h, from 8:00 in the morning until 4:00 in the afternoon, every other Tuesday for a period of 12 weeks to measure their fasting blood glucose levels.

### Glucose tolerance test (GTT)

GTTs were performed by injecting glucose intraperitoneally at a dose of 1 g/kg of body weight following a 16-h fasting period. Following glucose injection, blood glucose levels were measured at 0, 15, 30, 60, and 90 min.

### Insulin tolerance test (ITT)

ITTs were performed via intraperitoneally injection of insulin (0.75 U/kg of body weight) following an 8-h fasting period. Following the injection of insulin, blood glucose levels were measured at 0, 15, 30, 60, 90, 120 and 150 min.

### Transthoracic echocardiography

Transthoracic echocardiography was conducted when the mice reached the age of 20 weeks utilizing a high-resolution echocardiography system equipped with a 30-MHz high-frequency scanning head (VisualSonics Vevo770, VisualSonics, Toronto, Canada), as described previously [29]. The mice were anaesthetized with inhaled isoflurane (isoflurane, 1–1.5%) and placed supine on an imaging platform. Their paws were secured with ECG leads, and their chest fur was removed with Nair hair removal product. Ultrasonic coupling gel was then applied. M-mode and Doppler image loops of the left ventricle (LV) were obtained for 11–20 cardiac cycles, and at least three cycles per loop were averaged.

### Haemodynamic analysis

We utilized the conductance catheter technique to perform haemodynamic analysis, assessing both the systolic and diastolic function of the LV. Following previously established protocols, we evaluated the haemodynamics of mice at 20 weeks of age. Before sacrificing the mice, LV catheterization was carried out under anaesthesia. The right carotid artery was carefully isolated from the surrounding tissue during the procedure. Subsequently, we inserted a Millar Mikro-Tip catheter transducer (1.0-Fr, Millar 1.4F, SPR 835, Millar Instruments, Houston, TX) into the LV cavity via the same carotid artery. This transducer was linked to a pressure transducer from Millar Instruments (Houston, TX). Haemodynamic parameters were then recorded and analysed using PowerLab and LabChart software. At least three measurements were obtained for each parameter.

### Western blotting (WB) analysis

Frozen heart tissue and cultured cardiomyocytes were obtained and lysed at 4 ℃ for 30 min using radioimmunoprecipitation assay (RIPA) lysis buffer supplemented with protease and phosphatase inhibitor cocktails (NCM Biotech, Shanghai, China). The lysates were subsequently centrifuged at 12,000 × g for 15 min, and the resultant supernatant was utilized for Western blotting analysis. The protein concentrations were determined utilizing a bicinchoninic acid (BCA) protein assay kit (Beyotime Biotechnology, Shanghai, China). The homogenates were combined with sample loading buffer at a 1:1 ratio (v/v) and then boiled for 10 min. The protein samples were separated on a 12% SDS‒PAGE gel and then transferred onto polyvinylidene fluoride (PVDF) membranes (Millipore, Billerica, MA, USA). The membranes were blocked with 5% BSA solution for 2 h at room temperature. Subsequently, the membranes were incubated with primary antibodies overnight at 4 ℃. After overnight incubation, the membranes were washed with TBS-T solution. Next, the membranes were incubated with either HRP-conjugated goat anti-mouse or anti-rabbit secondary antibodies for 2 h at room temperature. Subsequently, the membranes were washed with TBS-T solution. The protein bands were visualized using ECL Plus chemiluminescence detection reagent (Beyotime Institute of Biotechnology, Nanjing, China). The density of each protein band was quantified using ImageJ.

### Antibodies for WB analysis

Anti-CAV3 (Abcam, ab289544, rabbit recombinant multiclonal, 1:1000), anti-NDUFA10 (Abcam, ab174829, rabbit monoclonal, 1:1000), anti-BAX (Abmart, M20008S, rabbit monoclonal, 1:1000), anti-Bcl2 (Abmart, T40056S, rabbit monoclonal, 1:1000), anti-cleaved caspase-3 (Cell Signalling, 9669 s, rabbit monoclonal, 1:1000), anti-SOD2 (ABclonal, A1340, rabbit monoclonal, 1:1000), anti-Cytochrome c (proteintech, 66264-1-Ig, mouse monoclonal, 1:5000), anti-VDAC1/Porin (proteintech, 55259-1-AP, rabbit polyclonal, 1:1000), anti-ATP1A1 (proteintech, 14418-1-AP, rabbit polyclonal, 1:5000), anti-Lamin B1 (proteintech, 12987-1-AP, rabbit polyclonal, 1:5000), anti-PDI (proteintech, 11245-1-AP, rabbit polyclonal, 1:500), anti-β-actin (ABclonal, AC026, rabbit monoclonal, 1:1000), anti-α-tubulin (Abcam, ab210797, mouse polyclonal, 1:1000), HRP-conjugated anti-rabbit or anti-mouse IgG (ABclonal, AS014, AS003, polyclonal, 1:5000), and HRP-conjugated anti-rabbit IgG- heavy chain or light chain (ABclonal, AS063, AS061, polyclonal, 1:5000) antibodies were used for WB analysis.

### RNA isolation and quantitative real-time- PCR (qPCR)

Total RNA was isolated from cells using TRIzol reagent (Invitrogen, Waltham, MA, USA) according to the manufacturer’s instructions. cDNA synthesis was performed by utilizing MultiScribe Reverse Transcriptase and total RNA (Thermo Fisher Scientific, Waltham, MA, USA). All primers were synthesized by AuGCT DNA-SYN (Wuhan, China) (Additional file 1: Table S1). The 7900HT Fast Real-Time PCR System was used for quantitative analysis of gene expression (Applied Biosystems, Foste, WA, USA). The data were analysed using the ΔΔCt method [30], and beta-actin RNA expression was utilized to normalize target mRNA expression.

### Morphological and histochemical analysis

Haematoxylin and eosin staining (H&E) (Servicebio, Wuhan, China) and wheat germ agglutinin (WGA) (Servicebio, Wuhan, China) staining of mouse cardiac tissues were formed to assess morphology according to standard methods. Sirius red staining was performed to assess the presence of myocardial fibrosis in cardiac tissue. Dihydroethidium (DHE) (Beyotime, Biotechnology, Shanghai, China), an oxidative fluorescence dye, and fluorescence microscopy were employed to assess in situ O2 generation in heart tissues. The fluorescence intensity was measured and analysed as previously described [29].

### Immunohistochemistry

CAV3 protein expression in mouse cardiac sections was assessed through immunohistochemical (IHC) staining using a DAB Detection Kit (Gene Tech, Shanghai, China) according to the manufacturer’s instructions (Abcam, ab289544).

### Immunofluorescence staining and confocal microscopy

To assess oxidative DNA damage, AC16 Cells were incubated with an anti-DNA/RNA Damage (8-oxoG) antibody (Abcam, ab62623), as described previously [31], and images were acquired using ZEN 2.1 software (Carl Zeiss, Oberkochen, Germany). Co-localization of DNA oxidation and mitochondria was assessed by immunofluorescent analysis of Translocase Of Outer Mitochondrial Membrane (TOM20, proteintech, 11802-1-AP) and 8-oxoG (Abcam, ab62623) in AC16 cells. To explore whether the place where CAV3 interacts with NDUFA10 is located in mitochondria, AC16 cells were labelled with the MitoTracker Red CMXRos probe (Thermo Fisher Scientific, Waltham, MA, USA), an anti-CAV3 antibody (Abcam, ab289544), an anti-NDUFA10 antibody (Santa Cruz, sc-376357), and DAPI (Servicebio, Wuhan, China). The cells were also incubated with an anti-Lamp1 antibody (Abcam, ab208943). Immunofluorescence co-localization images were captured using a laser scanning confocal microscope (Leica, SP8, Germany) and processed using LAS X 3.7.4 software (Leica, SP8, Germany).

### Transmission electron microscopy

Mitochondrial morphology in the LV of the heart was examined using transmission electron microscopy (TEM). Mitochondrial morphology and crista morphology were assessed using ImageJ (Madison, WI, USA), as previously described [32].

### Isolation of mitochondria and measurement of mitochondrial complex I activity

Mitochondria were isolated from freshly harvested mouse hearts, and the activity of mitochondrial complex I was assessed using a kit (Abbkin Scientific, Wuhan, China) according to the manufacturer’s instructions.

### Adenosine 5ʹ triphosphate (ATP) bioluminescence assay

An Enhanced ATP Assay Kit (Beyotime Biotechnology, Shanghai, China) was used to measure ATP content in cells and tissues according to the manufacturer’s instructions. ATP bioluminescence was promptly measured using a microplate luminometer (BioTek Inc., Synergy2, Vermount, USA) and standardized by each protein level.

### Analysis of apoptosis cells

Cardiomyocytes were stained with Annexin V-FITC and propidium iodide (PI) according to the instructions provided by the manufacturer of the Annexin V-FITC Apoptosis Detection Kit (Keygen Biotech, Nanjing, China). Flow cytometry (Beckman Coulter EPICS XL-MCL, Brea, CA, USA) was used to analyse apoptotic cells as previously described [33]. Apoptotic cells were detected in heart tissues through TUNEL staining following the manufacturer’s protocol (The Merck Group, Darmstadt, Germany). DAPI (Servicebio, Wuhan, China) was used to stain the nuclei of apoptotic cells. Images were acquired utilizing a laser scanning confocal microscope (Leica, SP8, Germany).

### Mitochondrial membrane potential assay

Following the manufacturer's instructions, a JC-1 assay kit (Beyotime Biotechnology, Shanghai, China) was used to measure the mitochondrial membrane potential. The red fluorescence intensity of JC-1 aggregates and the green fluorescence intensity of JC-1 monomers were measured using flow cytometry (Beckman Coulter EPICS XL-MCL, Brea, CA, USA).

### Cellular ROS assay

The levels of cellular ROS were measured using the CM-H2DCFDA dyes (MedChemExpress, New Jersey, USA) following the manufacturer's instructions, as described previously [34]. The fluorescence intensity of the cells was assessed using flow cytometry (Beckman Coulter EPICS XL-MCL, Brea, CA, USA).

### Mitochondrial ROS assay

The levels of mitochondrial ROS were measured using the mitochondrial superoxide indicator MitoSOX^™^ red (Invitrogen, Waltham, MA, USA) following the manufacturer's instructions and as previously described [32]. The fluorescence intensity of the cells was assessed using a confocal laser scanning microscope (Leica, SP8, Germany) and flow cytometry (Beckman Coulter EPICS XL-MCL, Brea, CA, USA).

### Mitochondrial oxygen consumption rate (OCR)

The OCR was measured using a Seahorse Extracellular Flux (XFe24) Analyzer (Agilent Seahorse Technologies, Shanghai, China) to monitor real-time changes in dissolved oxygen and proton concentrations in the cell culture medium, as previously described [32]. The assay was performed using the Wave software package provided with the Seahorse XF instrument following the manufacturer’s instructions. OCR measurements were normalized to protein level in each well.

### Cell transfection

Cardiomyocytes were transfected with a pcDNA3.1 plasmid containing the full-length human CAV3 gene, CAV3- or NDUFA10-specific small interfering RNA (siRNA), or shNDUFA10 (Additional file 1: Table S2) using Lipofectamine^™^ 2000 (Invitrogen, Shanghai, China) according to the manufacturer’s protocol. Transfection was conducted when the cells reached a confluence of 80% to 90%. After 4 h, the medium was replaced with complete medium. Subsequently, the cells were cultured under HGHF (33 mmol/L glucose and 250 μmol/L saturated free fatty acid palmitate) or NG for 24 h. The cells were harvested 48 h after transfection.

### Mitochondrial fractionation

Mitochondria were isolated from cardiomyocytes using a Cell Mitochondria Isolation Kit (C3601, Beyotime Biotechnology, Shanghai, China) according to the manufacturer’s instructions.

### Coimmunoprecipitation (Co-IP)

Co-IP assays were conducted using a previously described method [35]. Cells were lysed in RIPA lysis buffer containing a protease inhibitor cocktail. Mitochondria were isolated from cardiomyocytes. Proteins extracted from whole-cell lysates and mitochondrial fractions were used for Co-IP assays. After incubation with a rabbit anti-CAV3 antibody (Abcam, ab289544), rabbit anti-NDUFA10 antibody (Abcam, ab174829), or negative control IgG antibody (ABclonal, AC005) the protein-antibody complexes were subjected to immunoprecipitation using protein A/G agarose beads (Santa Cruz, CA, USA). The immunoprecipitates were washed with IP buffer, and the appropriate amounts of RIPA buffer and loading buffer were added before proceeding with subsequent experiments.

### LC‒MS/MS analysis

To identify proteins that interact with CAV3, coimmunoprecipitated proteins were electrophoresed and then visualized using Coomassie Brilliant blue R-250 staining (Beyotime Biotechnology, Shanghai, China) according to the manufacturer’s guidelines. The protein-antibody complexes, along with protein A/G agarose beads, were sent for LC‒MS/MS analysis to Spec-ally Life Technology (Wuhan, China).

### Statistical analysis

The data were analysed using GraphPad Prism 8.0 software (GraphPad Software, San Diego, CA, USA) and are presented as the mean ± standard deviation (SD). To compare normally distributed data between two groups, Student’s t test was employed. When there were more than two groups, one-way ANOVA followed by Tukey’s post hoc test for comparisons was conducted. Statistical significance was set at P < 0.05 for all analyses.

## Results

### CAV3 was decreased in diabetic mice hearts and knock-down CAV3 induced mitochondria-dependent intrinsic apoptosis in cardiomyocytes

**Fig. 1:**
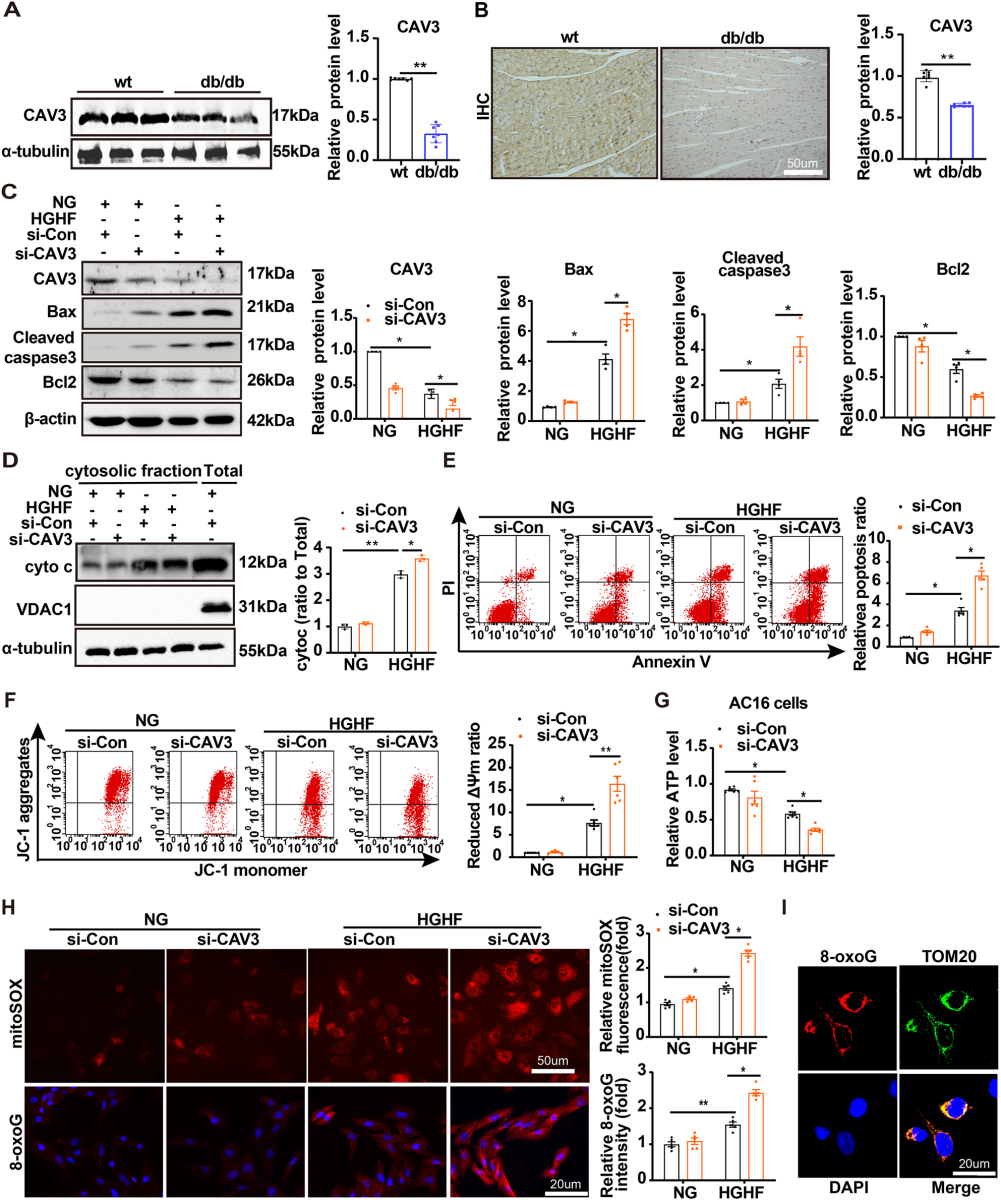
CAV3 was decreased in diabetic mice hearts and knock-down CAV3 induced mitochondria-dependent intrinsic apoptosis in cardiomyocytes. **A** Western blotting and associated quantitative analysis the CAV3 protein expression levels in the hearts of wt and db/db mice (n = 7 mice). **B** Representative immunohistochemical stains of CAV3 in the hearts of wt and db/db mice (n = 7 mice). **C** Representative Western blotting and quantitative analyses for CAV3, Bax, Cleaved caspase 3 and Bcl2 protein levels in cardiomyocytes treated with or without HGHF and with or without CAV3 downregulation (n = 4). **D** Representative Western blotting and quantitative analyses for the content of cytochrome C in cytoplasm treated with or without HGHF and with or without CAV3 downregulation (n = 3). **E** Flow cytometry and quantitative analysis of apoptosis cells by AnnexinV-FITC and propidium iodide (PI) staining in cardiomyocytes treated with or without HGHF and with or without CAV3 downregulation in vitro (n = 5). **F** Flow cytometry and quantification analysis of mitochondrial membrane potential by JC-1 in cardiomyocytes treated with or without HGHF and with or without CAV3 downregulation. High levels of green fluorescence (x-axis) represent reduced mitochondrial membrane potential (ΔΨm) and high levels of red fluorescence (y-axis) represents increased ΔΨm. A decrease in the red/green fluorescence is indicative of loss of ΔΨm (n = 6). **G** Normalized ATP production levels in cardiomyocytes treated with or without HGHF and with or without CAV3 downregulation in vitro (n = 6). **H** Representative confocal microscope of MitoSOX staining, immunofluorescence staining of 8-oxoG andquantitative analyses in cardiomyocytes treated with or without HGHF and with or without CAV3 downregulation (MitoSOX fluorescence, red; 8-oxoG fluorescence) (n = 5). **I** Immunofluorescence images of 8-oxoG and TOM20 in the AC16 cells treated with HGHF. AC16 cells were stained with 8-oxoG antibodies (Alexa Fluor 594), DAPI, and TOM20 (Alexa Fluor 488). Data are depicted as mean ± SD. *p < 0.05, **p < 0.01

Western blotting and immunohistochemical staining showed that there was a notable decrease in the expression of CAV3 in db/db mice hearts compared to wt mice (Fig. 1A, B), and the same effects were observed in cardiomyocytes cultured under HGHF conditions (Additional file 1: Figure S1A). Our findings demonstrated that exposure to HGHF conditions significantly increased Bax and cleaved caspase 3 expression, reduced Bcl2 expression (Fig. 1C), increased the content of cytochrome C in cytoplasm (Fig. 1D), aggravated the proportion of apoptotic cells (Fig. 1E) and altered the mitochondrial membrane potential in cardiomyocytes (Fig. 1F), and all these effects were exacerbated by CAV3 knockdown (Fig. 1C–F). Additionally, decreased CAV3 expression significantly aggravated the HGHF-induced decrease in ATP levels (Fig. 1G) and ROS level (Fig. 1H, I and Additional file 1: Figure S1B).

These results demonstrated that CAV3 expression was decreased in the hearts of diabetic mice and that CAV3 knockdown induced mitochondria-dependent intrinsic apoptosis in cardiomyocytes.

### CAV3 overexpression protected against cardiac dysfunction and cardiac remodeling in db/db mice

**Fig. 2:**
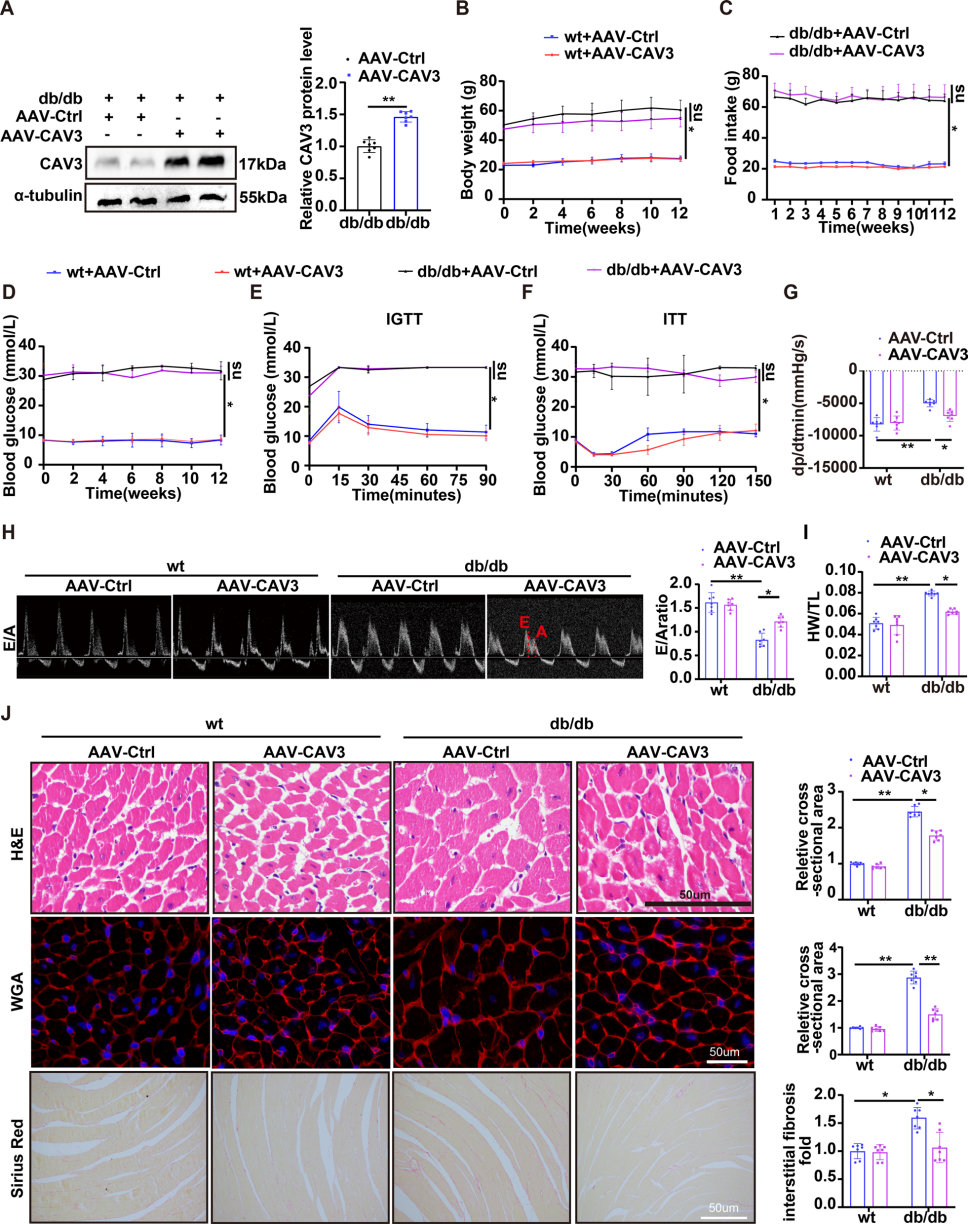
Overexpression of CAV3 protected against cardiac dysfunction and cardiac remodeling in db/db mice. **A** Western blotting and associated quantitative analysis the CAV3 protein expression levels in the hearts of db/db mice with or without CAV3 overexpression in vivo (n = 7 mice). **B**–**F** The body weight, food intake and blood glucose levels of wt and db/db mice among each group were showed by a line chart (n = 7 mice). **G** Hemodynamic analysis (dp/dtmin) of wt mice and db/db mice with or without CAV3 overexpression. (n = 7 mice). **H** Representative images of echocardiograph (E/A) and relevant quantification analysis in wt mice and db/db mice with or without CAV3 overexpression (n = 7 mice). **I** The HW/TL ratio was quantitatively analyzed in wt mice and db/db mice with or without CAV3 overexpression (n = 7 mice). **J** Representative images of H&E staining and WGA staining to detect cardiomyocyte cross-sectional area, Sirius red staining to detect myocardial interstitial collagen and relevant quantification analysis in wt mice and db/db mice with or without CAV3 overexpression (n = 7 mice). Data are depicted as mean ± SD. *p < 0.05, **p < 0.01

To explore the function of CAV3 in DCM in db/db mice, AAV9 combined with the cTnT promoter delivery system was injected into mice to overexpress CAV3. Western blotting showed a notable increase in CAV3 expression in the cardiac tissue of AAV9-CAV3-treated mice (Fig. 2A and Additional file 1: Figure S2). Our data showed no significant changes in heart rate (HR), left ventricular ejection fraction (LVEF), left ventricular fractional shortening (LVFS) or maximum peak derivative of pressure over time (dp/dt) among the groups (Additional file 1: Table S3). Body weight, food intake, and blood glucose levels were significantly increased in db/db mice, but we did not find differences in these measures between the AAV9-Ctrl and AAV9-CAV3-treated mice (Fig. 2B–F). The decreases in the minimum dp/dt and early mitral diastolic wave/late mitral diastolic wave (E/A) ratio indicated that db/db mice exhibited exacerbated cardiac dysfunction (Fig. 2G, H), as well as an increased heart weight/tibia length (HW/TL) (Fig. 2I), myocardium size and worse myocardial fibrosis (Fig. 2J). Interestingly, CAV3 overexpression significantly alleviated these changes (Fig. 2G–J).

These data showed the protective effects of CAV3 overexpression against cardiac dysfunction and cardiac remodeling in db/db mice.

### CAV3 overexpression prevented mitochondrial dysfunction in diabetic cardiomyocytes in vivo and in vitro models

**Fig. 3:**
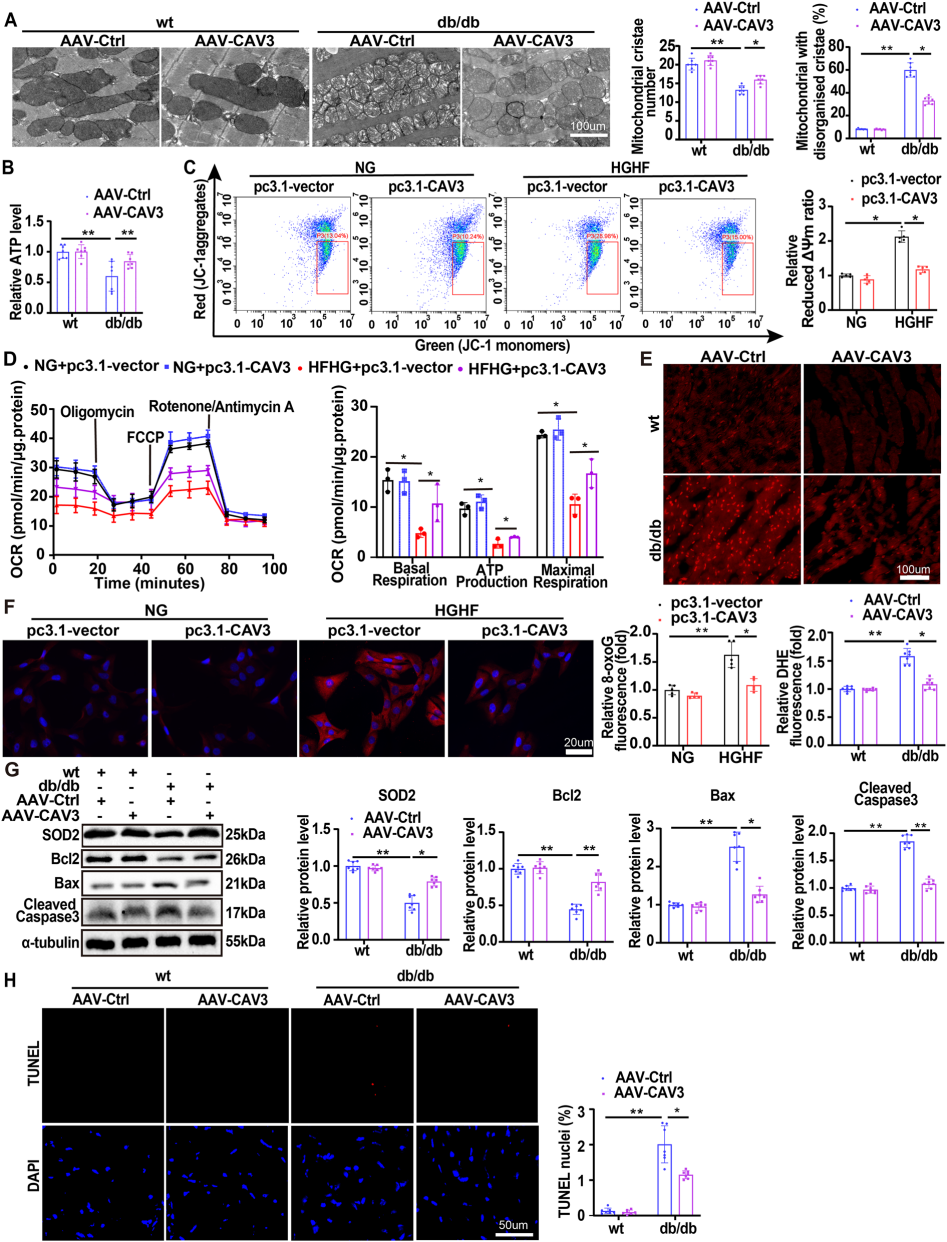
Overexpression of CAV3 prevented mitochondrial dysfunction in diabetic cardiomyocytes in vivo and in vitro models. **A** Representative transmission electron microscope images of cardiac mitochondria and quantification analysis of mitochondrial cristae number and the proportion of mitochondria with disorganized cristae in wt mice and db/db mice with or without CAV3 overexpression (n = 7 mice). **B** Normalized ATP production levels in cardiomyocytes of wt mice and db/db mice with or without CAV3 overexpression (n = 7 mice). **C** Flow cytometry analysis and quantification of mitochondrial membrane potential by JC-1 in cardiomyocytes treated with or without HGHF and with or without CAV3 overexpression. (n = 5). **D** Extracellular flux analysis of OCRs and respective quantitative analysis in cardiomyocytes treated with or without HGHF and with or without CAV3 overexpression (n = 3). **E** Representative images of DHE staining and quantitative analysis of ROS levels in the hearts of wt mice and db/db mice with or without CAV3 overexpression (n = 7 mice). **F** Representative images of immunofluorescence staining of 8-oxoG and quantitative analyses in cardiomyocytes treated with or without HGHF and with or without CAV3 overexpression (8-oxoG fluorescence, red) (n = 5). **G** Representative Western blotting and quantitative analyses for SOD2, Bcl2, Bax and Cleaved caspase 3 protein levels in cardiomyocytes of wt mice and db/db mice with or without CAV3 overexpression (n = 7 mice). **H** TUNEL assay by double staining with TUNEL (red) and DAPI (blue) detected apoptotic cells in mice hearts and quantitative analysis of wt mice and db/db mice with or without CAV3 overexpression (n = 7 mice). Data are depicted as mean ± SD. *p < 0.05, **p < 0.01

Mitochondria morphology observed by TEM was significantly improved by AAV9-CAV3 treatment compared to AAV9-Ctrl group in db/db mice hearts (Fig. 3A). According to the data shown in Fig. 3B, cardiac tissues from db/db mice treated with AAV9-CAV3 exhibited a higher ATP content than those from the AAV9-Ctrl group (Fig. 3B). Consistently, similar results were observed in HGHF-exposed cardiomyocytes (Additional file 1: Figure S3A). JC-1 staining and OCR assays demonstrated that CAV3 overexpression reversed the HGHF-induced changes in the mitochondrial membrane potential (Fig. 3C) and mitochondrial respiratory function in cardiomyocytes (Fig. 3D). There was a significant increase in ROS level (DHE staining, Fig. 3E) in db/db mice hearts and HGHF-exposed cardiomyocytes (8-oxoG, CM-H2DCFDA and MitoSox staining, Fig. 3F and Additional file 1: Figure S3B-C), which was attenuated by CAV3 overexpression. Furthermore, Western blotting showed that CAV3 overexpression significantly increased recombinant superoxide dismutase 2 (SOD2), Bcl2 protein levels and decreased Bax, cleaved caspase 3 protein levels in both HGHF-exposed cardiomyocytes and db/db mice hearts (Fig. 3G and Additional file 1: Figure S3D). The content of cytochrome C in cytoplasm was also reduced by CAV3 overexpression (Additional file 1: Figure S3E). Additionally, similar results were observed by Annexin V-FITC/PI apoptosis staining (Additional file 1: Figure S3F) in cardiomyocytes and TUNEL staining (Fig. 3H) in db/db mice hearts.

These data showed that CAV3 overexpression prevented mitochondrial dysfunction.

### CAV3 interacted with mitochondrial complex I by anchoring NDUFA10 and maintaining its stability

**Fig. 4:**
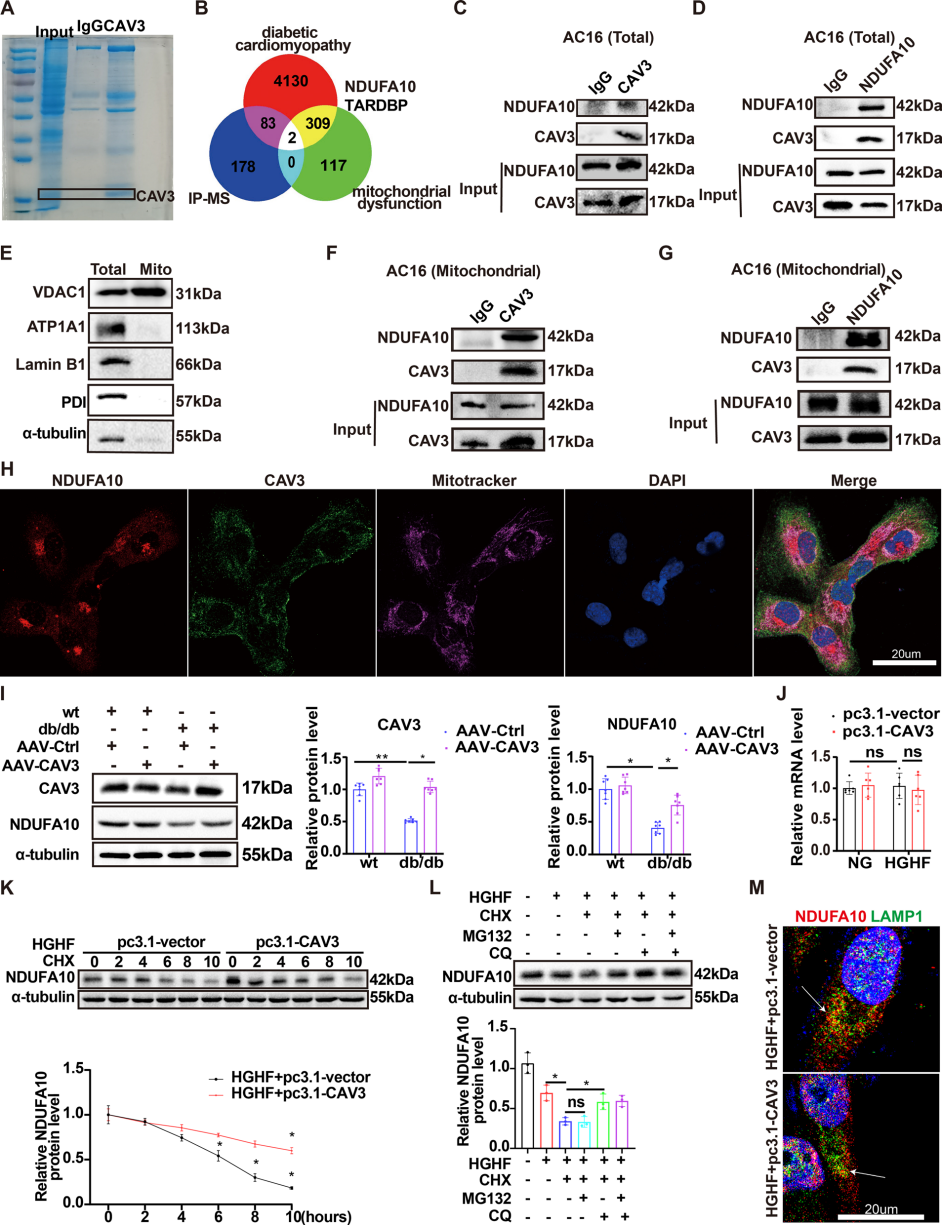
CAV3 interacted with mitochondrial complex I by anchoring NDUFA10 and maintaining its stability. **A** IP assay was carried out using CAV3 antibody and Coomassie Brilliant blue was stained to indicate CAV3 bands. **B** Through bioinformatics methods, NDUFA10 and TARDBP were overlapped from the LC–MS/MS analysis, DCM-related Genes (DisGeNet & Gencards) and Mitochondrial dysfunction-related genes (GeneCards). **C**, **D** IP assay was carried out using CAV3 or NDUFA10 antibody in cardiomyocytes. **E** Representative Western blotting assessing the purity of the mitochondria isolated. **F**–**G** IP assay was carried out using CAV3 or NDUFA10 antibody in mitochondria isolated from AC16 cells. **H** Immunofluorescence images of cardiomyocytes were stained with mitochondria-targeting dye (Mito-Tracker, Deep Red, Alexa Fluor 647), DAPI, NDUFA10 antibodies (Alexa Fluor 594), and CAV3 antibodies (Alexa Fluor 488). **I** Western blotting and quantitative analyses protein levels in cardiomyocytes of wt mice and db/db mice with or without CAV3 overexpression (n = 7 mice). **J** The levels of NDUFA10 mRNA in cardiomyocytes treated with or without HGHF and with or without CAV3 overexpression (n = 6). **K** CAV3 plasmid (pcDNA3.1-CAV3) was transfected into cardiomyocytes using Lipofectamine 2000 and treated with HGHF. The transfected cells were treated with 100 µmol/L cycloheximide for 0, 2, 4, 6, 8, 10 h. The protein levels of NDUFA10 were shown by a line chart (n = 3). **L** Cardiomyocytes were treated with or without HGHF, CHX (100 µmol/L), and MG132 (10 µmol/L) or CQ (50 µmol/L) for 24 h. Quantitative analyses of NDUFA10 protein levels among each group (n = 3). **M** Immunofluorescence images of NDUFA10 and lysosomes-targeting dye (Lamp1) in the cardiomyocytes treated with or without CAV3 overexpression. Cardiomyocytes were stained with NDUFA10 antibodies (Alexa Fluor 594), DAPI, and Lamp1 (Alexa Fluor 488). Data are depicted as mean ± SD. *p < 0.05, **p < 0.01

To further explore the mechanism underlying the role of CAV3 in DCM, we conducted IP assays using an anti-CAV3 antibody. Coomassie brilliant blue staining revealed a strong CAV3 band near 17 kDa (Fig. 4A). LC‒MS/MS analysis identified 263 enriched proteins. The proteins NDUFA10 and TARDBP were selected for further study using bioinformatics methods (Fig. 4B). Co-IP assays showed that the anti-CAV3 antibody pulled down NDUFA10 but not TARDBP (Fig. 4C, D and Additional file 1: Figure S4A–C). Next, we detected whether the place where CAV3 interacts with NDUFA10 is located in mitochondria. We extracted mitochondria for Co-IP assay. Figure 4E verified the purity of the mitochondria isolated was high. The results showed that anti-CAV3 antibody or anti-NDUFA10 could pull down each other too (Fig. 4F, G). Moreover, immunofluorescence staining demonstrated the colocalization of CAV3 and NDUFA10 in the mitochondria of HGHF-exposed cardiomyocytes (Fig. 4H), confirming that NDUFA10 might be a putative target of CAV3 involved in the regulation of mitochondrial function.

The structural stability and proper functioning of the oxidative phosphorylation (OXPHOS) system are dependent on the integrity of mitochondrial complex I, which is not only the largest but also the most complicated enzyme complex of this system [36, 37]. NDUFA10, a subunit of mitochondrial complex I, is responsible for the assembly of complex I [37–39]. Western blotting showed a significant decrease in NDUFA10 protein levels in cardiomyocytes exposed to HGHF conditions and db/db mice hearts, which was reversed by CAV3 overexpression (Additional file 1: Figure S5 and Figure 4I). Interestingly, the NDUFA10 mRNA level was not changed after CAV3 overexpression (Fig. 4J). These data indicated that the regulatory effect of CAV3 on the NDUFA10 protein might be related to posttranscriptional processes.

To determine the mechanism by which NDUFA10 is regulated by CAV3, cycloheximide (CHX) chase assay is used in cardiomyocytes cultured under HGHF conditions. The data showed that NDUFA10 protein levels gradually decreased as the duration of CHX treatment increased, but this decrease was significantly delayed after overexpression of CAV3 (Fig. 4K). In addition, our findings showed that the lysosome inhibitor chloroquine (CQ), but not the proteasome inhibitor MG132, restored NDUFA10 protein levels (Fig. 4L). Furthermore, immunofluorescence staining demonstrated that NDUFA10 colocalized with LAMP1 in cardiomyocytes. However, this colocalization within lysosomes was considerably attenuated after CAV3 overexpression (Fig. 4M). These data verified that the influence of CAV3 on NDUFA10 degradation might be regulated by lysosomal pathway.

### CAV3 overexpression restored mitochondrial function partially relied on NDUFA10 in vivo and in vitro models

**Fig. 5:**
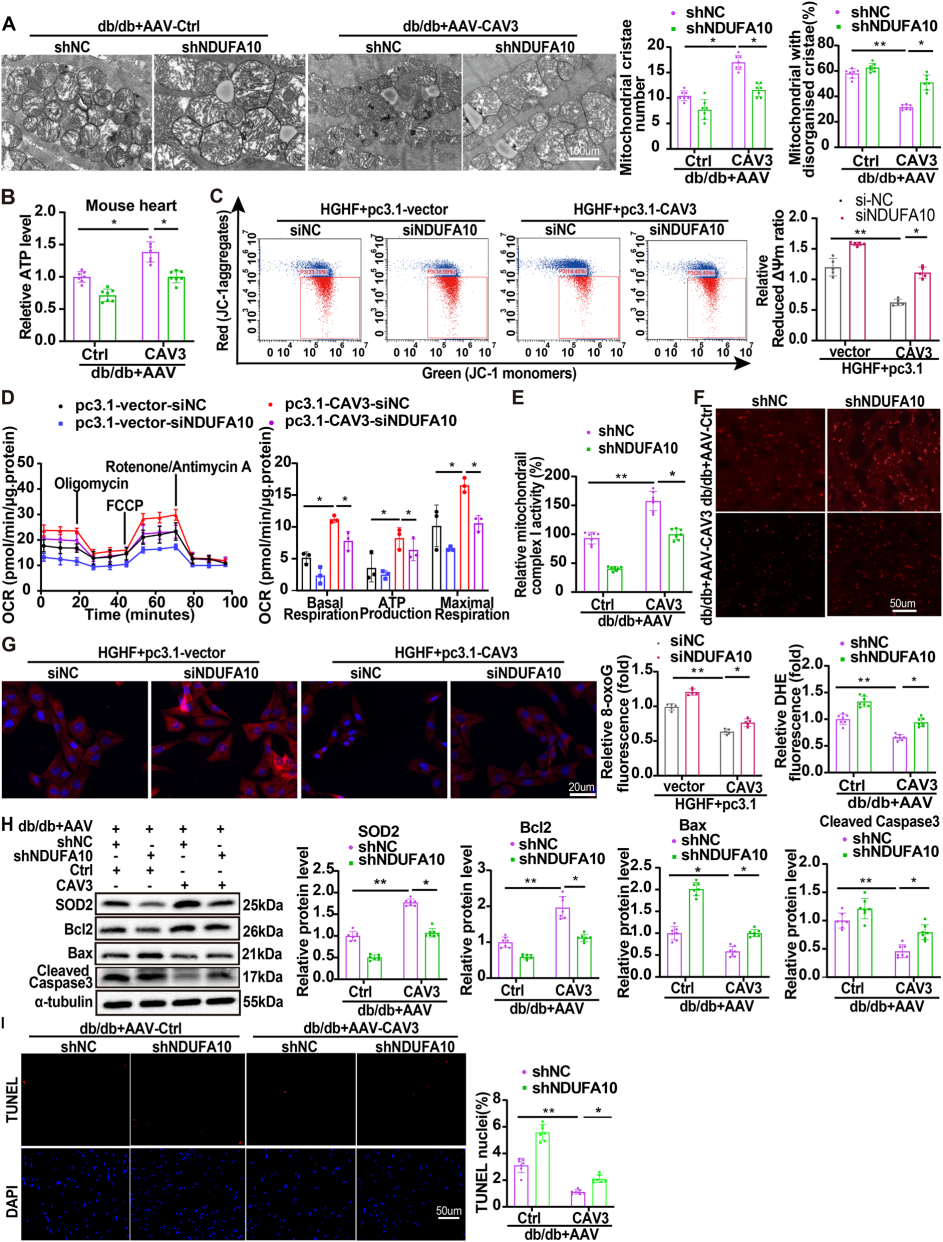
CAV3 overexpression restored mitochondrial function partially relied on NDUFA10 in vivo and in vitro models. **A** Representative transmission electron microscope images of cardiac mitochondria and quantification analysis of mitochondrial cristae number and the proportion of mitochondria with disorganized cristae in different db/db mice groups (n = 7 mice). **B** Normalized ATP production levels in cardiomyocytes of db/db mice transfected with or without AAV-CAV3 or AAV-shNDUFA10 (n = 7 mice). **C** Flow cytometry and quantitative analysis of mitochondrial membrane potential by JC-1 in cardiomyocytes treated with HGHF and transfected with or without pc3.1-CAV3 or siNDUFA10 (n = 5). **D** Extracellular flux analysis of OCRs and respective quantitative analysis in cardiomyocytes treated with HGHF and transfected with or without pc3.1-CAV3 or siNDUFA10 (n = 3). **E** The activity of mitochondrial complex I in cardiomyocytes of db/db mice transfected with or without AAV-CAV3 or AAV-shNDUFA10 (n = 7 mice). **F** Representative images of DHE staining and quantitative analysis of ROS levels in the hearts of db/db mice transfected with or without AAV-CAV3 or AAV-shNDUFA10 (n = 7 mice). **G** Representative images of immunofluorescence staining of 8-oxoG and quantitative analyses in cardiomyocytes treated with HGHF and transfected with or without pc3.1-CAV3 or siNDUFA10 (8-oxoG fluorescence, red) (n = 5). **H** Representative Western blotting and quantitative analyses for SOD2, Bcl2, Bax and Cleaved caspase 3 protein levels in cardiomyocytes of db/db mice transfected with or without AAV-CAV3 or AAV-shNDUFA10 (n = 7 mice). **I** TUNEL assay by double staining with TUNEL (red) and DAPI (blue) detected apoptotic cells in the hearts and quantitative analysis of different db/db mice groups (n = 7 mice). Data are depicted as mean ± SD. *p < 0.05, **p < 0.01

To further explore whether the protective effects of CAV3 overexpression depended on the regulatory function of NDUFA10, AAV9-CAV3 and AAV-shNDUFA10 were used to increase CAV3 and knock down NDUFA10 expression (Additional file 1: Figure S6A, B). CAV3 overexpression significantly increased the number of mitochondrial cristae and decreased the proportion of mitochondria with disorganized cristae in db/db mice, while these effects were partially attenuated by downregulation of NDUFA10 (Fig. 5A). Cardiac tissues from db/db mice treated with AAV9-CAV3-shNC had a higher ATP content than those treated with AAV9-Ctrl-shNC (Fig. 5B). However, this effect was partially attenuated by downregulation of NDUFA10. Consistently, similar results were observed in HGHF-exposed cardiomyocytes (Additional file 1: Figure S7A). JC-1 staining and OCR assays also demonstrated that downregulation of NDUFA10 partially attenuated the protective effect of CAV3 overexpression on the mitochondrial membrane potential (Fig. 5C) and mitochondrial respiratory function in HGHF-exposed cardiomyocytes (Fig. 5D). In addition, CAV3 overexpression increased mitochondrial complex I activity in cardiac tissue from db/db mice, but this protective effect was also partially attenuated by downregulation of NDUFA10 (Fig. 5E). Similar effects were observed for ROS levels (DHE staining, Fig. 5F) in db/db mouse hearts and HGHF-exposed cardiomyocytes (8-OxoG, CM-H2DCFDA and MitoSox staining, Fig. 5G and Additional file 1: Figure S7B, C). Furthermore, Western blotting demonstrated that NDUFA10 downregulation partially prevented the CAV3 overexpression induced protein expression changes, such as the increases in SOD2 and Bcl2 levels and reduction in Bax and cleaved caspase 3 levels in both HGHF-exposed cardiomyocytes and db/db mouse hearts (Fig. 5H and Additional file 1: Figure S7D). The content of cytochrome C in cytoplasm reduced by CAV3 overexpression was partially reversed after NDUFA10 knock down (Additional file 1: Figure S7E). Additionally, Annexin V-FITC/PI apoptosis staining in cardiomyocytes (Additional file 1: Fig. S7F) and TUNEL staining (Fig. 5I) in db/db mice hearts also showed that downregulation of NDUFA10 partially attenuated the protective effect of CAV3 overexpression.

These results suggested that CAV3 upregulation restored mitochondrial function partially through NDUFA10.

### Cardiac-restricted NDUFA10 expression partially attenuated CAV3 benefits in diabetic cardiomyopathy

**Fig. 6:**
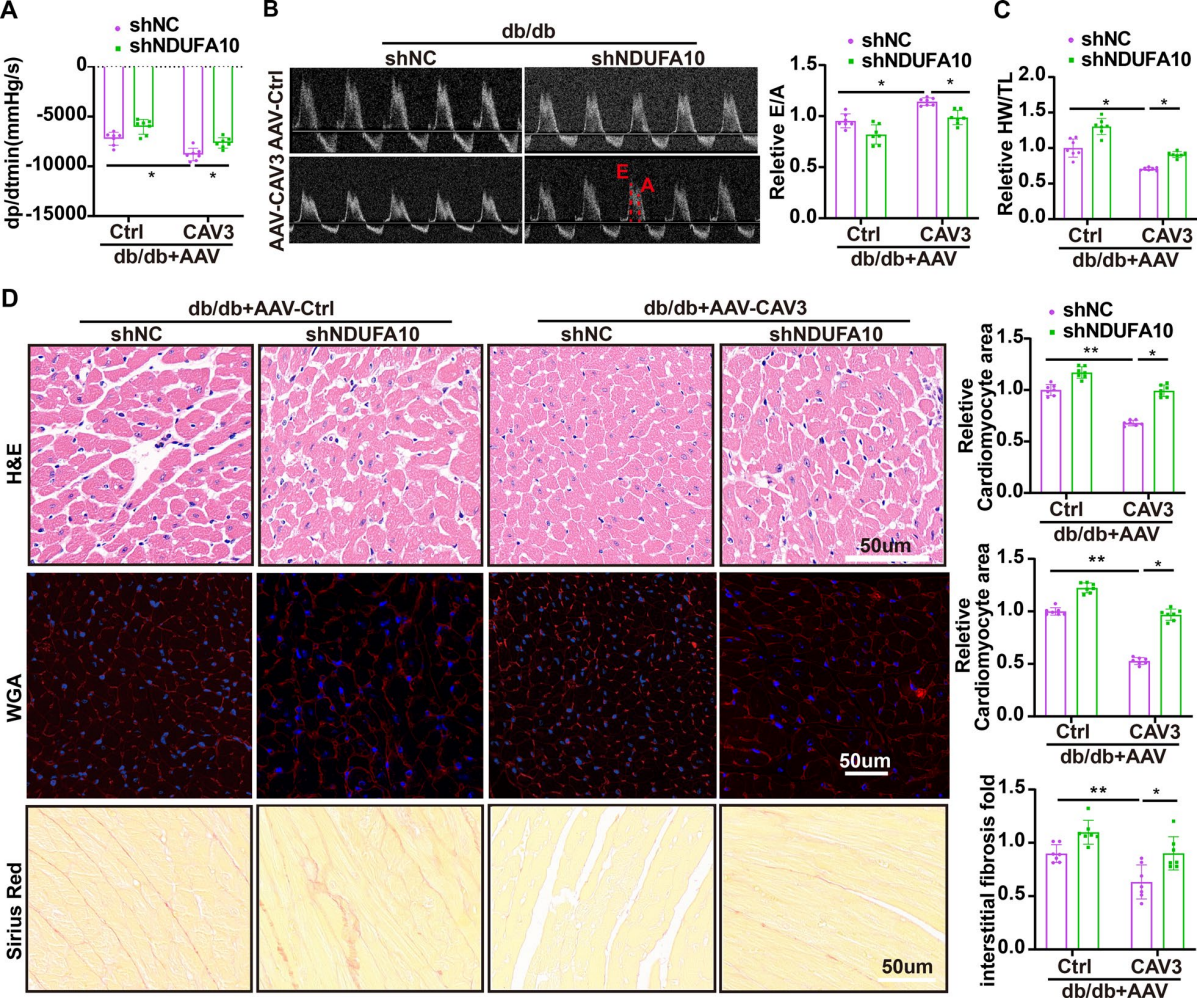
Cardiac-restricted NDUFA10 expression partially attenuated CAV3 benefits in diabetic cardiomyopathy. **A** Hemodynamic analysis (dp/dtmin) of db/db mice transfected with or without AAV-CAV3 or AAV-shNDUFA10 (n = 7 mice). **B** Representative images of echocardiograph (E/A) and relevant quantification analysis in db/db mice transfected with or without AAV-CAV3 or AAV-shNDUFA10 (n = 7 mice). **C** The HW/TL ratio was quantitatively analyzed in db/db mice transfected with or without AAV-CAV3 or AAV-shNDUFA10 (n = 7 mice). **D** Representative images of H&E staining and WGA staining to detect cardiomyocyte cross-sectional area, Sirius red staining to detect myocardial interstitial collagen and relevant quantification analysis in db/db mice transfected with or without AAV-CAV3 or AAV-shNDUFA10 (n = 7 mice). Data are depicted as mean ± SD. *p < 0.05, **p < 0.01

There were no significant changes in HR, LVEF, LVFS or maximum dp/dt (Additional file 1: Table S4) among the groups. Interestingly, CAV3 overexpression significantly alleviated cardiac dysfunction (Fig. 6A, B), and decreased the HW/TL (Fig. 6C), myocardium size and myocardial fibrosis (Fig. 6D) in db/db mice. However, these effects were partially reversed by the downregulation of NDUFA10 in the AAV9-CAV3-shNDUFA10-treated db/db mice (Fig. 6A–D). These findings suggested that CAV3 overexpression had a protective effect against DCM through a mechanism partially dependent on NDUFA10.

## Discussion

**Fig. 7:**
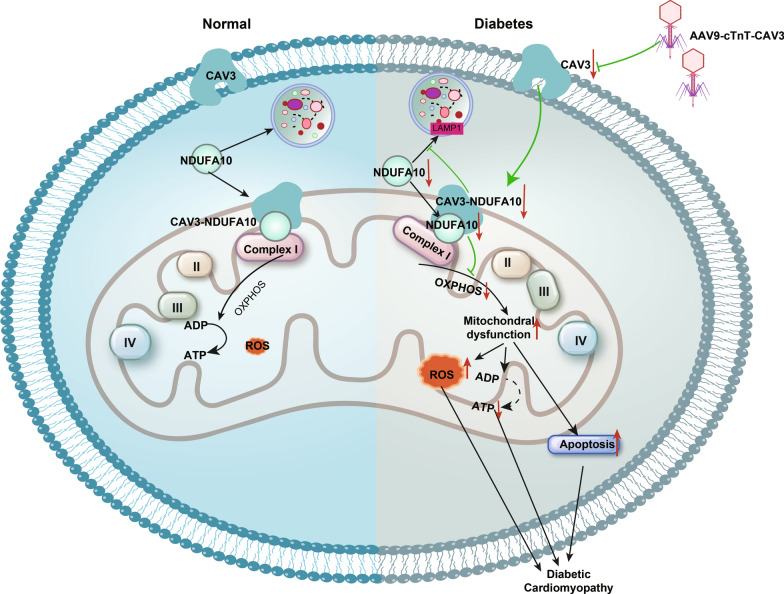
The schematic representation of the mechanisms by which CAV3 downregulation exacerbates diabetic cardiomyopathy via inducing NDUFA10-mediated mitochondrial dysfunction in diabetes. CAV3 expression is decreased in cardiomyocytes under diabetic conditions. Downregulation of CAV3 leads to the downregulation of NDUFA10, which is degraded via the lysosomal pathway. Consequently, the reduction of NDUA10 in mitochondria lead to a decrease in the mitochondrial complex I activity, resulting in mitochondrial dysfunction. As a result, ATP production decreases, mitochondrial ROS levels increase and cell apoptosis occurs, thereby leading to cardiac dysfunction

This study revealed that hearts of db/db mice exhibited significant downregulation of CAV3, which resulted in mitochondrial dysfunction. Furthermore, we demonstrated that CAV3 overexpression protected against DCM of in db/db mice by alleviating mitochondrial dysfunction, reducing ROS production, and attenuating cardiomyocyte apoptosis. Mechanistically, we found that CAV3 overexpression helped maintain the stability of NDUFA10 by reducing its degradation through the lysosomal pathway. Additionally, we further revealed that the protective effect of CAV3 overexpression against DCM was achieved via upregulation of NDUFA10, an important subunit of complex I, which improved mitochondrial function. Specifically, we discovered that CAV3 overexpression alleviated DCM induced by mitochondrial dysfunction by reducing the degradation of NDUFA10 through the lysosomal pathway (Fig. 7). Therefore, targeting CAV3 to alleviate mitochondrial dysfunction could be a potential therapeutic approach for DCM.

CAV3 is abundantly expressed in cardiomyocytes and is vital for various cellular functions [40]. Previous studies have demonstrated that animals with global CAV3 ablation exhibit defective insulin signalling and impaired glucose uptake and utilization [41–44]. Our study revealed that cardiac-specific overexpression of CAV3 improved cardiac function in db/db mice but had no effect on plasma glucose levels. It is hypothesized that cardiac-specific overexpression of CAV3 may have no impact on overall metabolism. Previous studies have suggested that a high blood glucose level is not the only factor that drives DCM progression indiabetic patients [45, 46]. Consistently, our results indicated that progression of DCM was not solely dependent on elevated blood glucose levels. Some studies have also suggested that intensive glucose and lipid control strategies may not fully relieve DCM [47–49], suggesting that the development of DCM may be influenced by other factors or comorbidities. A growing number of studies have indicated that the progression of DCM is significantly influenced by mitochondrial dysfunction [50–52]. A previous study showed that alleviating mitochondrial dysfunction can have a protective effect against DCM [11]. Our recent study revealed that CAV3 overexpression in the heart offers protection against cardiac dysfunction and remodelling in db/db mice by improving mitochondrial function.

Db/db mice have been widely used as an animal model of diabetic cardiomyopathy [3, 53]. In our study, we observed a reduction in cardiac diastolic function in db/db mice, while systolic function remained unchanged. These findings are consistent with prior research [54] and confirm that diastolic dysfunction is an early hallmark of DCM and that patients present with a normal ejection fraction before exhibiting cardiac systolic dysfunction with a reduced ejection fraction. The variations in cardiac function in db/db mice observed among different studies may have been due to differences in the timing and manner of the intervention.

Mitochondrial complex I consists of a total of 45 subunits along with 15 assembly factors [55]. The absence of NDUFA10, a vital subunit of mitochondrial complex I, leads to functional deficiency of the complex, which results in mitochondrial impairment [56]. Previous studies have also found an association between NDUFA10 and several diseases, such as nonalcoholic steatohepatitis [57], type 2 diabetes [58], Alzheimer's disease [59] and Leigh disease [38]. According to a previous study, decreased levels of NDUFA10 protein impair mitochondrial function, increase mitochondrial ROS production, and ultimately lead to disruption of cardiac structure and function [60]. Therefore, targeting NDUFA10 to alleviate mitochondrial dysfunction could emerge as a potential therapeutic approach for DCM. Previous study demonstrated that overexpression of CAV3 improved mitochondrial dysfunction in myocardial ischemia/reperfusion injury through activation of the Akt signaling pathway [61]. Li H, et al. demonstrated that increasing CAV3 expression activated STAT3 and consequently improved postischemic cardiac mitochondrial function and attenuated myocardial injury [62]. Notably, there is no reported relationship between NDUFA10 and CAV3. However, a crucial discovery from our current investigation is that the protective effect of CAV3 against DCM partially depends on the regulation of NDUFA10. Further validation is needed to determine whether the AKT signaling pathway and STAT3 signaling pathway play roles in Cav3-regulated improvement of mitochondrial function in DCM.

This observation was corroborated by the fact that downregulation of NDUFA10 in the diabetic heart partially attenuated the protective effect of CAV3 overexpression both in vitro and in vivo. In agreement with previous studies [36], our investigation demonstrated that NDUFA10 protein levels decrease in DCM via the lysosomal pathway. Moreover, overexpression of CAV3 maintains the stability of NDUFA10 by reducing its degradation, thereby restoring the activity of mitochondrial complex I, improving the efficiency of the respiratory chain, reducing the production of mitochondrial ROS, and ultimately enhancing cardiac function in db/db mice.

This study discusses the novel finding of the protective effect of CAV3 on DCM and explores how CAV3 protects the myocardium from mitochondrial damage through inhibition NDUFA10 degradation, thereby adding a new theoretical perspective to the pathogenesis of DCM. CAV3 and NDUFA10 may emerge as new therapeutic targets for preventing and treating DCM. In addition, CAV3 is uniquely expressed in myocytes, and utilizing gene therapy for treating DCM holds the advantage of fewer side effects and greater developmental prospects in the future. However, this study has certain limitations. First, the determination of CAV3 expression in the cardiac tissue of diabetic patients was hindered by ethical concerns in our study. Second, we did not utilize cardiomyocyte-specific CAV3-knockout mice in our study. Moreover, we suggested that CAV3 restoration with AAV therapy might be a potential approach in diabetic hearts, whereas the translational conception has not been verified in non-human primates. Moreover, the human sample size was lacking for CAV3 expression and correlation analysis. Additionally, we focus largely on type 2 diabetes induced DCM and whether and how CAV3 in type 1 diabetes associated heart injury needs further examination. Furthermore, Considering CAV3 is primarily localized to membrane structures such as the plasma membrane and mitochondrial membrane, whereas NDUFA10 is mainly expressed in mitochondria, we just detected their interaction in mitochondria, but not plasma membrane. Finally, although we provided AAV9-CAV3 as a potential strategy, specific agonists targeting CAV3 deserve to be further investigated.

Despite the limitations mentioned above, our current study provides compelling evidence that downregulation of CAV3 promotes mitochondrial dysfunction and DCM progression. Additionally, CAV3 overexpression alleviates DCM by maintaining NDUFA10 stability, reducing degradation through the lysosomal pathway, improving mitochondrial function, reducing ROS production, and decreasing cardiomyocyte apoptosis. Our study provides valuable insights into the involvement of mitochondrial dysfunction in DCM, highlighting the potential of restoring CAV3 levels in the treatment of DCM.

### Supplementary Information

**Additional file 1. Table S1**. Primers sequence.** Table S2**. Knockdown sequence.** Table S3**. Heart dimensions andfunction.** Table S4**. Heart dimensions and function.** Figure S1**. CAV3 expression was decreased in cardiomyocytes with HGHF treatment and downregulation of CAV3 aggravated cellular ROS production in vitro models.** Figure S2**. CAV3 expression was notable increase in the cardiac tissue of AAV9-CAV3-treated mice.** Figure S3**. Overexpression of CAV3 attenuated the decrease of ATP levels, ROS production and cardiomyocyte apoptosis in vitro models.** Figure S4**. CAV3 interacts with NDUFA10 but not TARDBP.** Figure S5**. CAV3 overexpression reversed the decreasing of NDUFA10 protein levels in vitro models.** Figure S6**. CAV3 expression notably increased and NDUFA10 expression notably decreased in the cardiac tissue of AAV9-CAV3 and AVV9-shNDUFA10-treated mice.** Figure S7**. CAV3 overexpression attenuating the decrease of ATP levels, ROS production and cardiomyocyte apoptosis partially depended on NDUFA10 regulation in vitro models.

## Data Availability

The data that support the findings of this study are available from the corresponding author upon reasonable request.The DCM-related Genes data have been deposited in the DisGeNet & Gencards database (https://www.disgenet.org/, https://www.genecards.org/). The Mitochondrial dysfunction-related genes data have been deposited in the GeneCards (https://www.genecards.org/). The mass spectrometry proteomics data have been deposited to the ProteomeXchange Consortium (http://proteomecentral.proteomexchange.org) via the iProX partner repository with the dataset identiffer IPX0006941000.

## Abbreviations

DCM: Diabetic cardiomyopathy
CAV3: Caveolin 3
ROS: Reactive oxygen species
LC–MS/MS: Liquid chromatography tandem mass spectrometry
HGHF: High-glucose and high-fat
WB: Western blotting
IHC: Immunohistochemical
AAV: Adeno-associated virus
wt: Wild-type
HR: Heart rate
LVEF: Left ventricular ejection fraction
LVFS: Left ventricular fractional shorting
dp/dt: Derivative of pressure over time
E/A ratio: Early mitral diastolic wave/late mitral diastolic wave ratio
H&E: Hematoxylin and eosin staining
WGA: Wheat germ agglutinin
HW/TL: Ratio of heart weight to tibia length
OCR: Oxygen consumption rate
SOD2: Superoxide dismutase 2
TUNEL: Terminal deoxynucleotidyl transferase-mediated nick end labeling
CO-IP: Co-immunoprecipitation
CHX: Cycloheximide
CQ: Chloroquine
Lamp1: Lysosomal associated membrane protein 1
AVV9-shNDUFA10: Adeno-associated virus carrying shNDUFA10
qPCR: Quantitative Real-time PCR
pc3.1-CAV3: CAV3 plasmid
siCAV3: CAV3-specific small interfering RNA
siNDUFA10: NDUFA10-specific small interfering RNA
shNDUFA10: NDUFA10-specific short hairpin RNA
