# Supplementary material for: CAV3 alleviates diabetic cardiomyopathy via inhibiting NDUFA10-mediated mitochondrial dysfunction
Source: J Transl Med. 2024 Apr 26;22:390. doi: 10.1186/s12967-024-05223-6 (PMC11055322; doi:10.1186/s12967-024-05223-6)

**CAV3** **alleviates** **diabetic cardiomyopathy via** **inhibiting NDUFA10-mediated mitochondrial dysfunction**

Ping Guo^1,2#^, Shuiqing Hu^1,2#^, Xiaohui Liu^1,2^, Miaomiao He^1,2^, Jie Li^1,2^, Tingqiong Ma^1,2^, Man Huang^1,2^, Qin Fang^1,2*^ and Yan Wang^1,2*^

^1^Division of Cardiology and Department of Internal Medicine, Tongji Hospital, Tongji Medical College, Huazhong University of Science and Technology, Wuhan 430030, China

^2^Hubei Key Laboratory of Genetics and Molecular Mechanisms of Cardiological Disorders, Wuhan 430030, China

# Ping Guo and Shuiqing Hu contributed equally to this work.

**Corresponding authors:**

Dr. Yan Wang, M.D., Ph.D.

Division of Cardiology, Department of Internal Medicine, Tongji Hospital, Tongji Medical College, Huazhong University of Science and Technology

E-Mail: [newswangyan@tjh.tjmu.edu.cn](mailto:newswangyan@tjh.tjmu.edu.cn)

Dr. Qin Fang, M.D., Ph.D.

Division of Cardiology, Department of Internal Medicine, Tongji Hospital, Tongji Medical College, Huazhong University of Science and Technology

E-Mail: fangqin140716@126.com

**This file includes:**

Supplementary Table 1-4

Supplementary Figure legends

Supplementary Figure 1-7

**Supplementary material**

**Major resources tables**

**Supplementary Table 1. primers sequence**

| Name | Sense | Antisense |
| --- | --- | --- |
| NDUFA10 (human) | TCTGCAAGGGAAGCTCAAGA | GGCCCGGCAGAGTAATCG |
| β-actin (human) | TGACGTGGACATCCGCAAAG | CTGGAAGGTGGACAGCGAGG |

**Supplementary Table 2. Knockdown sequence**

| Name | Sense | Antisense |
| --- | --- | --- |
| si-CAV3 | GCGUGUGGAAGGUGAGCUATT | UAGCUCACCUUCCACACGCTT |
| si-NDUFA10 | GGUGGUAGAGGACAUUGAATT | UUCAAUGUCCUCUACCACCTT |
| siNDUFA10 (shNDUFA10) | TCTTGGTGATAAGACAACCAA | TTGGTTGTCTTATCACCAAGA |

siNDUFA10 (shNDUFA10) represents the siNDUFA10 sequence used to construct shNDUFA10.

**Supplementary Table 3. Heart dimensions and function**

| **Groups/ mice** | **wt-Ctrl** | **wt-CAV3** | **db/db-Ctrl** | **db/db-CAV3** |
| --- | --- | --- | --- | --- |
| HR (bpm) | 454.44 ± 25.51 | 453.03 ± 41.03 | 471.88 ± 34.52 | 462.87 ± 33.96 |
| LVEF (%) | 79.85 ± 2.45 | 81.48 ± 2.17 | 75.38 ± 2.54 | 76.35 ± 2.14 |
| LVFS (%) | 41.09 ± 3.16 | 41.05 ± 2.07 | 39.24 ± 3.17 | 41.88 ± 2.27 |
| LVAWd (mm) | 0.85 ± 0.14 | 0.86 ± 0.16 | 0.81 ± 0.11 | 0.83 ± 0.10 |
| LVAWs (mm) | 1.45 ± 0.30 | 1.52 ± 0.13 | 1.38 ± 0.16 | 1.50 ± 0.23 |
| LVPWd (mm) | 0.82 ± 0.17 | 0.86 ± 0.13 | 0.75 ± 0.13 | 0.77 ± 0.17 |
| LVPWs (mm) | 1.87 ± 0.16 | 2.09 ± 0.12 | 1.86 ± 0.18 | 1.95 ± 0.19 |
| LVIDd (mm) | 3.40 ± 0.27 | 3.20 ± 0.31 | 3.88 ± 0.32* | 3.52 ± 0.33 |
| LVIDs (mm) | 1.84 ± 0.21 | 1.85 ± 0.19 | 1.91 ± 0.23 | 1.88 ± 0.15 |
| dp/dtmax (mmHg/s) | 8938.20 ± 1140.12 | 9001.27 ± 1537.09 | 7429.38 ± 767.52 | 8683.02 ± 1531.18 |

All values are depicted as mean ± SD. Statistical analysis was done using One-Way-ANOVA, **P*<0.05 vs wt-Ctrl.

**Supplementary Table 4. Heart dimensions and function**

| **Groups/db/db** | **Ctrl-shNC** | **Ctrl-**  **shNDUFA10** | **CAV3-shNC** | **CAV3-shNDUFA10** |
| --- | --- | --- | --- | --- |
| HR (bpm) | 398.59 ± 58.15 | 383.06 ± 51.84 | 395.40 ± 49.04 | 369.23 ± 69.24 |
| LVEF (%) | 76.98 ± 3.80 | 76.69 ± 4.04 | 78.15 ± 3.8 | 77.61 ± 2.35 |
| LVFS (%) | 39.44 ± 4.05 | 39.28 ± 2.70 | 42.38 ± 5.89 | 41.23 ± 4.31 |
| LVIDd (mm) | 3.82 ± 0.43 | 4.18 ± 0.17 | 3.82 ± 0.31 | 3.85 ± 0.29 |
| LVIDs (mm) | 1.94 ± 0.09 | 1.98 ± 0.13 | 1.90 ± 0.12 | 1.94 ± 0.16 |
| LVPWd (mm) | 0.76 ± 0.16 | 0.74 ± 0.16 | 0.80 ± 0.15 | 0.77 ± 0.11 |
| LVPWs (mm) | 2.01 ± 0.12 | 1.97 ± 0.12 | 2.07 ± 0.19 | 1.96 ± 0.25 |
| LVAWd (mm) | 0.83 ± 0.20 | 0.80 ± 0.13 | 0.84 ± 0.17 | 0.83 ± 0.10 |
| LVAWs (mm) | 1.40 ± 0.29 | 1.38 ± 0.31 | 1.55 ± 0.29 | 1.38 ± 0.27 |
| dp/dtmax (mmHg/s) | 7155.57 ± 399.09 | 7152.43 ± 457.13 | 7932.09 ± 637.25 | 7330.17 ± 733.60 |

All values are depicted as mean ± SD. Statistical analysis was done using One-Way-ANOVA, *P*<0.05.

**Supplementary figures and figure legends**

**Supplementary figure legends**

**Supplementary Figure 1. CAV3 expression was decreased in cardiomyocytes with HGHF treatment and downregulation of CAV3 aggravated cellular ROS production in vitro models**

(A) Western blotting and associated quantitative analysis the CAV3 protein levels in cardiomyocytes treated with or without HGHF (n = 6).

(B) Flow cytometry after CM-H2DCFDA staining and quantification analysis of cellular ROS production levels in cardiomyocytes treated with or without HGHF and with or without CAV3 downregulation in vitro (n=5).

**Supplementary Figure 2. CAV3 expression was notable increase in the cardiac tissue of AAV9-CAV3-treated mice**

Western blot analysis to evaluate the expression of CAV3 in different tissues.

**Supplementary Figure 3. Overexpression of CAV3 attenuated the decrease of ATP levels, ROS production and cardiomyocyte apoptosis in vitro models**

(A) Normalized ATP production levels in cardiomyocytes treated with or without HGHF and with or without CAV3 overexpression in vitro (n=6).

(B) Flow cytometry after CM-H2DCFDA staining and quantification analysis of cellular ROS production levels in cardiomyocytes treated with or without HGHF and with or without CAV3 overexpression in vitro (n=5).

(C) Flow cytometry after MitoSOX staining and quantification analysis of mitochondria ROS production levels in cardiomyocytes treated with or without HGHF and with or without CAV3 overexpression in vitro (n=5).

(D) Representative Western blotting and quantitative analyses for SOD2, Bcl2, Bax and Cleaved caspase 3 protein levels in cardiomyocytes treated with or without HGHF and with or without CAV3 overexpression in vitro (n=3).

(E) Representative Western blotting and quantitative analyses for the content of cytochrome C in cytoplasm treated with or without HGHF and with or without CAV3 downregulation (n=3).

(F) Flow cytometry of apoptosis by AnnexinV-FITC and propidium iodide (PI) staining and quantification analysis of apoptotic cells treated with or without HGHF and with or without CAV3 overexpression in vitro (n=5).

Data are depicted as mean ± SD. *p < 0.05, **p < 0.01.

**Supplementary Figure 4.** **CAV3 interacts with NDUFA10 but not TARDBP.**

(A) IP assay was carried out using CAV3 or TARDBP antibody in cardiomyocytes.

(B-C) The LC-MS/MS spectrum of CAV3 (EIDLVNRDPK) (B) and NDUFA10 (LLQYSDALEHLLTTGQGVVLER) (C).

**Supplementary Figure 5. CAV3 overexpression reversed the decreasing of NDUFA10 protein levels in vitro models**

Western blotting and quantitative analyses protein levels in cardiomyocytes treated with or without HGHF and with or without CAV3 overexpression in vitro.

**Supplementary Figure 6. CAV3 expression notably increased and NDUFA10 expression notably decreased in the cardiac tissue of AAV9-CAV3 and AVV9-shNDUFA10-treated mice**

(A) Western blotting and quantification analysis of NDUFA10 expression in HL1 cells transfected with shRNA-NDUFA10 (n=3).

(B) Western blotting and associated quantitative analysis the CAV3 and NDUFA10 protein levels in the hearts of db/db mice transfected with or without AAV-CAV3 or AAV-shNDUFA10 (n=7 mice).

**Supplementary Figure 7. CAV3 overexpression attenuating the decrease of ATP levels, ROS production and cardiomyocyte apoptosis partially depended on NDUFA10 regulation in vitro models**

(A) Normalized ATP production levels in cardiomyocytes treated with HGHF and transfected with or without pc3.1-CAV3 or siNDUFA10 (n=6).

(B) Flow cytometry after CM-H2DCFDA staining and quantification analysis of cellular ROS production levels in cardiomyocytes treated with HGHF and transfected with or without pc3.1-CAV3 or siNDUFA10 (n=5).

(C) Flow cytometry after MitoSOX staining and quantification of mitochondria ROS production levels in cardiomyocytes treated with HGHF and transfected with or without pc3.1-CAV3 or siNDUFA10 in vitro (n=5).

(D) Representative Western blotting and quantitative analyses for SOD2, Bcl2, Bax and Cleaved caspase 3 protein levels in cardiomyocytes treated with HGHF and transfected with or without pc3.1-CAV3 or siNDUFA10 in vitro (n=3).

(E) Representative Western blotting and quantitative analyses for the content of cytochrome C in cytoplasm treated with HGHF and transfected with or without pc3.1-CAV3 or siNDUFA10 in vitro (n=3).

(F) Flow cytometry of apoptosis by AnnexinV-FITC and propidium iodide (PI) staining and quantification analysis of apoptotic cells treated with HGHF and transfected with or without pc3.1-CAV3 or siNDUFA10 in vitro (n=5).

Data are depicted as mean ± SD. *p < 0.05, **p < 0.01.

**Supplementary figures**


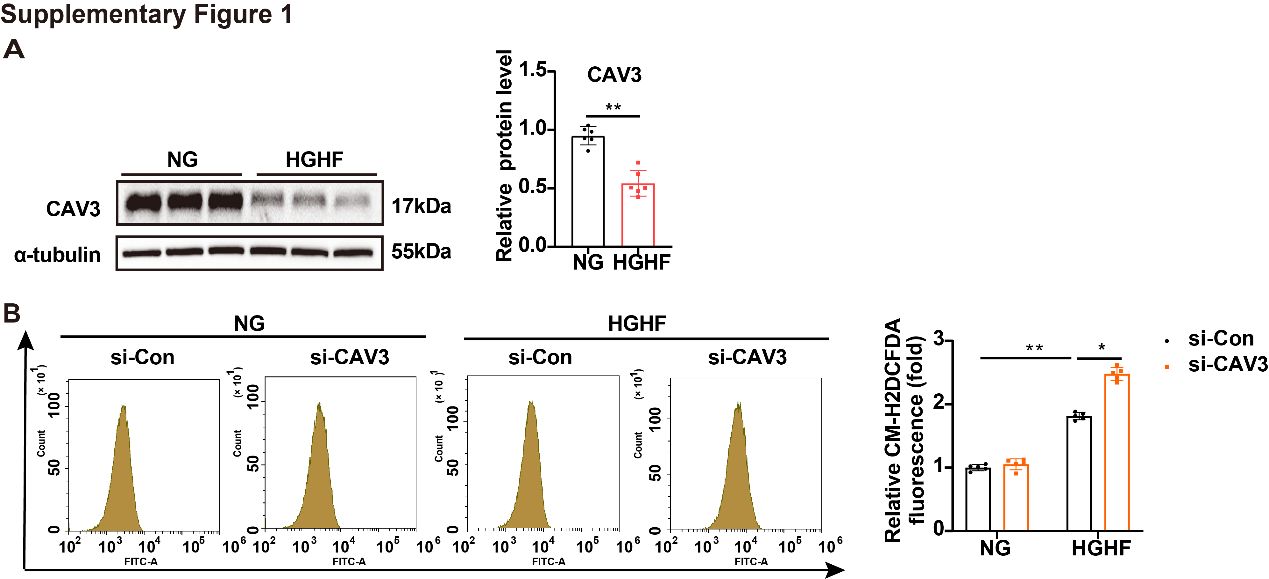


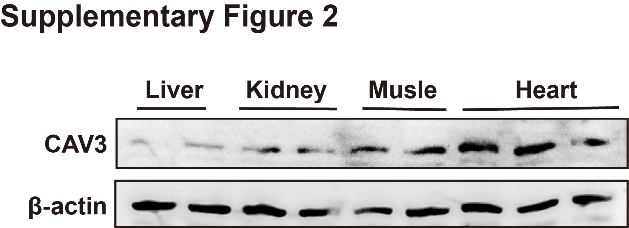


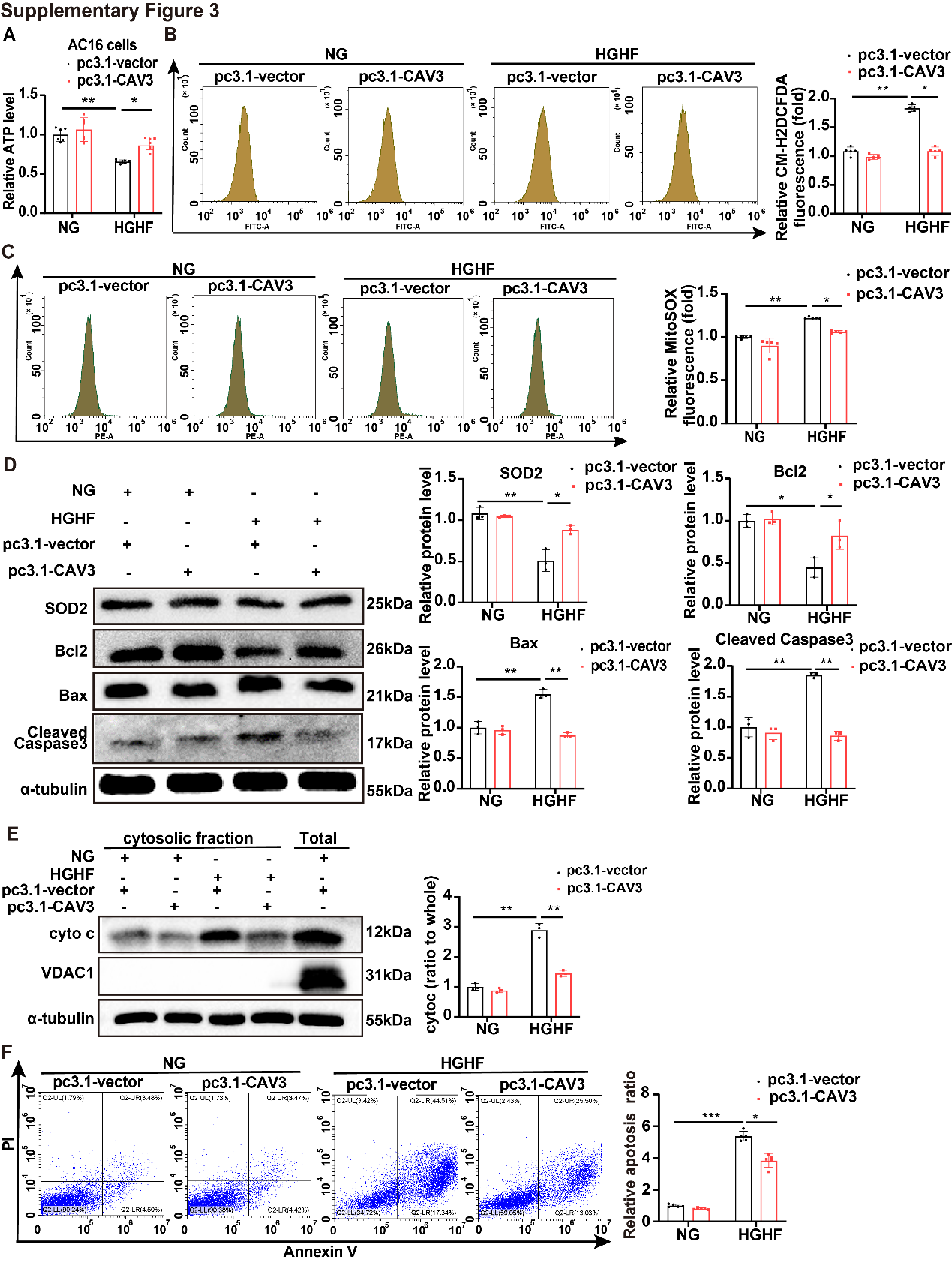


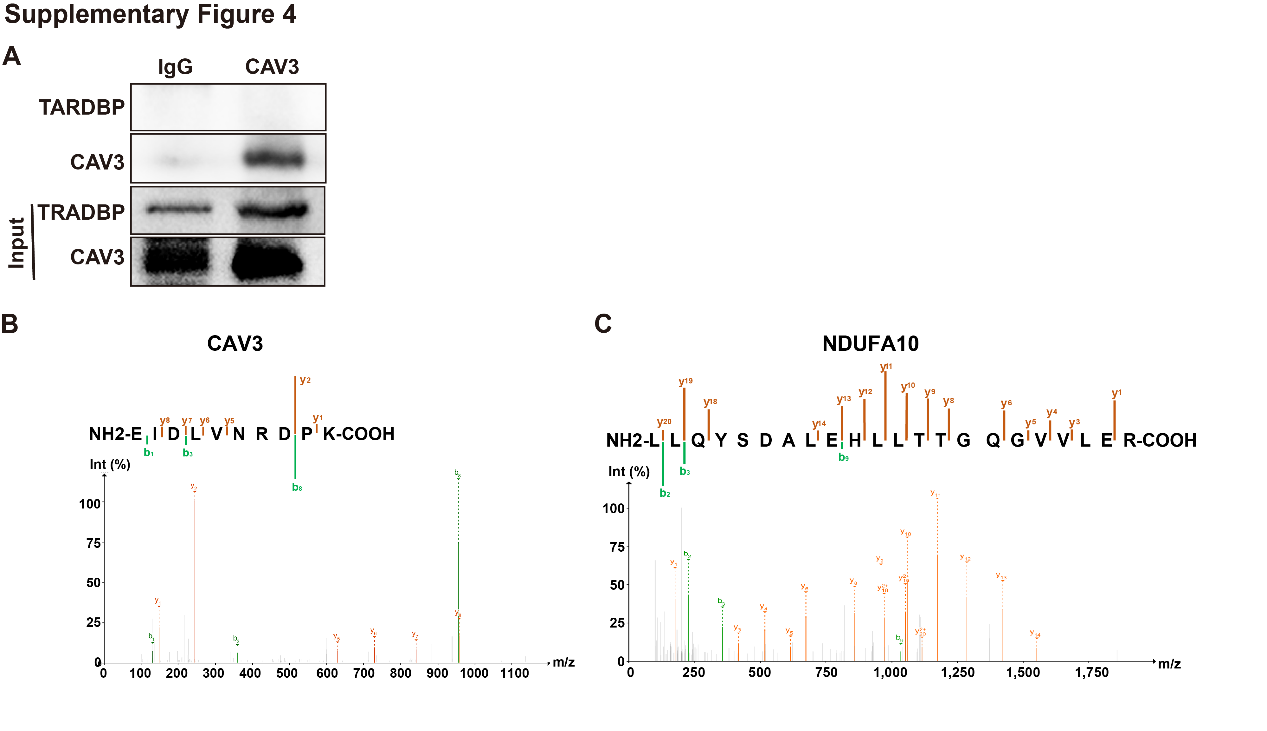


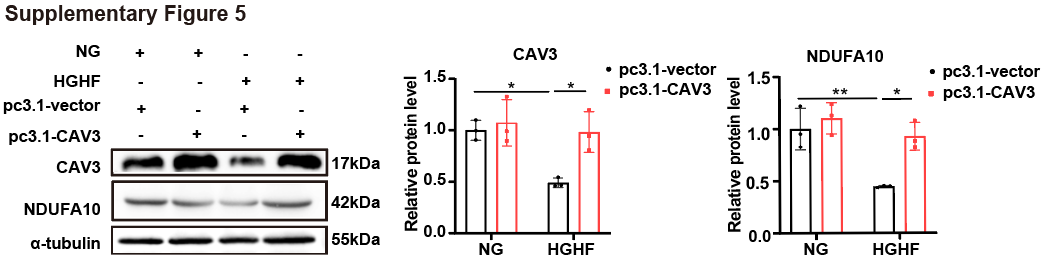


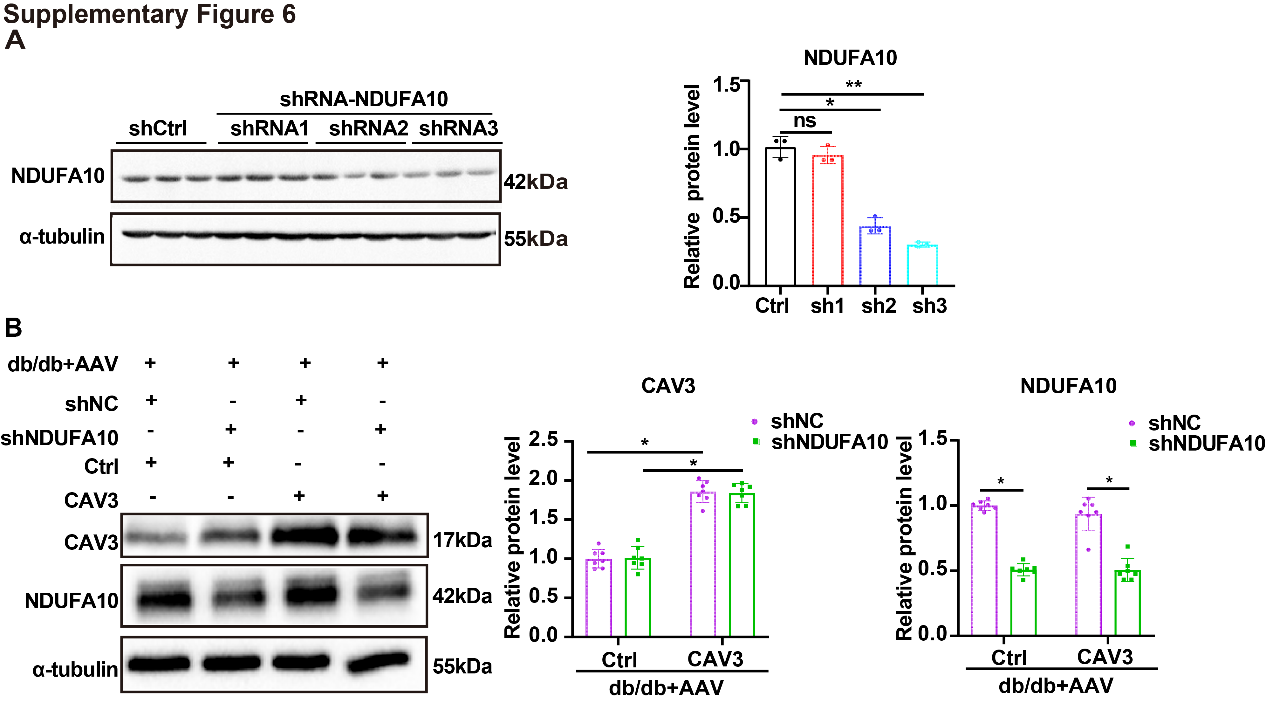


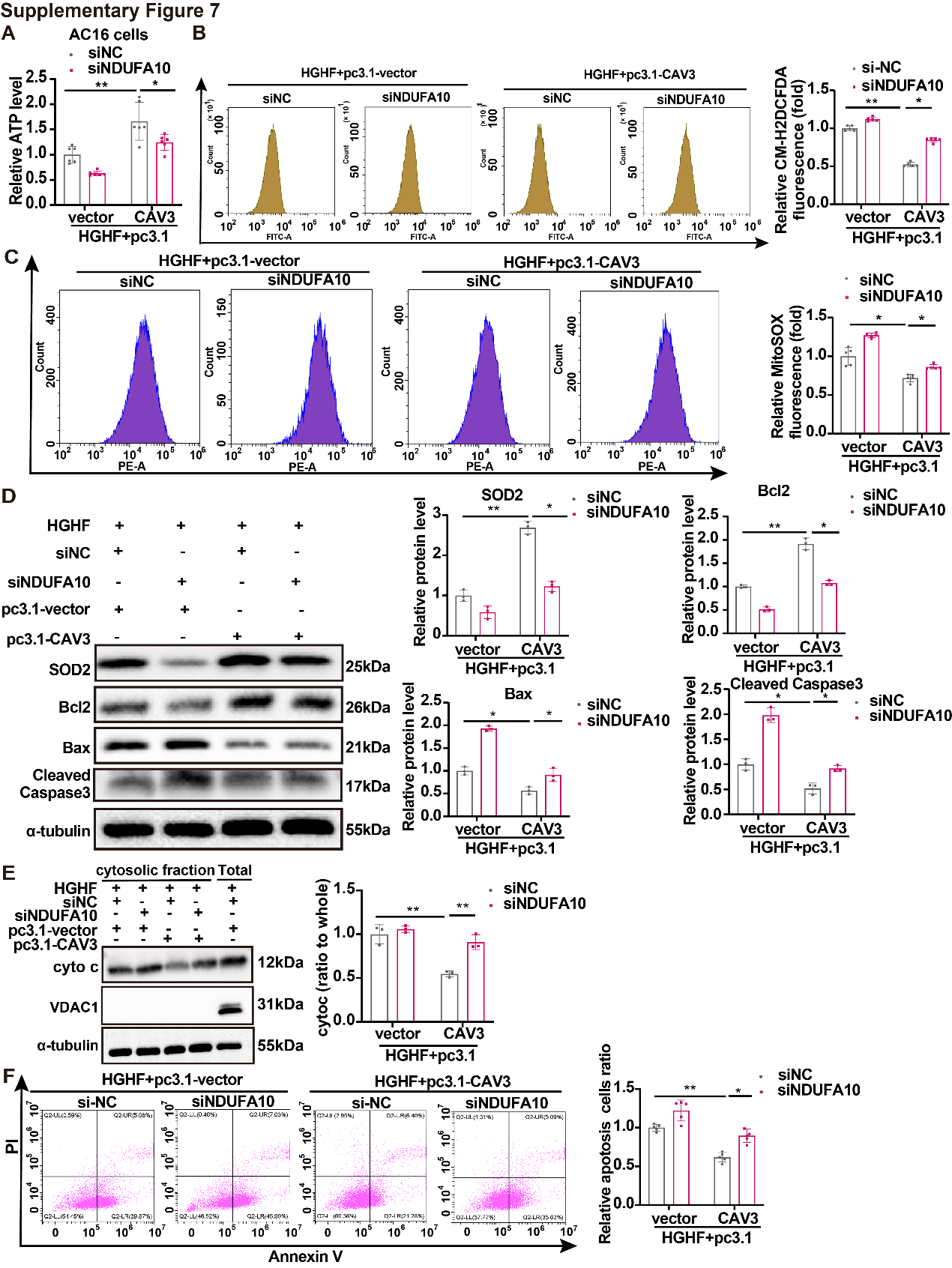

Supplement: Supplementary file 1 — Additional file 1. Table S1. Primers sequence. Table S2. Knockdown sequence. Table S3. Heart dimensions andfunction. Table S4. Heart dimensions and function. Figure S1. CAV3 expression was decreased in cardiomyocytes with HGHF treatment and downregulation of CAV3 aggravated cellular ROS production in vitro models. Figure S2. CAV3 expression was notable increase in the cardiac tissue of AAV9-CAV3-treated mice. Figure S3. Overexpression of CAV3 attenuated the decrease of ATP levels, ROS production and cardiomyocyte apoptosis in vitro models. Figure S4. CAV3 interacts with NDUFA10 but not TARDBP. Figure S5. CAV3 overexpression reversed the decreasing of NDUFA10 protein levels in vitro models. Figure S6. CAV3 expression notably increased and NDUFA10 expression notably decreased in the cardiac tissue of AAV9-CAV3 and AVV9-shNDUFA10-treated mice. Figure S7. CAV3 overexpression attenuating the decrease of ATP levels, ROS production and cardiomyocyte apoptosis partially depended on NDUFA10 regulation in vitro models. [file 12967_2024_5223_MOESM1_ESM.docx]
